# Prospective, Randomized, Comparative Study of the Cutaneous Effects of a Topical Body Treatment Compared to a Bland Moisturizer

**DOI:** 10.1093/asj/sjab161

**Published:** 2021-04-02

**Authors:** Jean Carruthers, Gyasi Bourne, Michaela Bell, Alan Widgerow

**Affiliations:** 1 Carruthers Instruments Inc., Vancouver, British Columbia, Canada; 2 Alastin Skincare, Inc., Carlsbad, CA, USA

## Abstract

**Background:**

Over time human skin thins and loses elasticity; topical treatments attempt to reverse this process.

**Objectives:**

The aim of this study was to assess the efficacy of TransFORM Body Treatment (TFB) in skin rejuvenation compared to a bland moisturizer on the extensor and volar forearms.

**Methods:**

Blinded participants were given 2 products to apply on the designated forearms with follow-up at 4, 8, and 12 weeks. Measurements included skin thickness, photography, dermatopathology, cutaneous elasticity determined by 2 different methods, and patient-reported outcomes. All were compared to baseline.

**Results:**

Changes between bland moisturizer and TFB were recorded for the following parameters. (1) Roughness: extensor –0.09 mm for bland moisturizer and –0.26 mm for TFB (*P* = 0.174); volar 0.01 mm for bland moisturizer and –0.23 mm for TFB (*P* = 0.004). (2) Recoil velocity: volar –56°/sec for bland moisturizer and –24°/sec for TFB (*P* = 0.61); extensor –95°/sec for bland moisturizer and –63°/sec for TFB (*P* = 0.57). Retraction speed: volar –3.25 ms for bland moisturizer and –20.08 ms for TFB (*P* = 0.33); extensor –2.17 ms for bland moisturizer and –10.83 ms for TFB (*P* = 0.66). Histologically, TFB resulted in an increase in mucopolysaccharide content, new collagen, and number of elastin fibers in the papillary dermis. Changes in the Rao-Goldman score were also observed: volar –0.17 for bland moisturizer and –0.33 for TFB (*P* = 0.25); extensor –0.08 for bland moisturizer and –0.17 for TFB (*P* = 0.36).

**Conclusions:**

Histology showed production of new collagen and elastin. Quantification of changes in skin thickness, skin retraction speed, and skin recoil velocity showed trends that agree with the visual data.

**Level of Evidence: 4:**

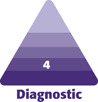

Skin is a living fabric and over time human skin thins and loses its elasticity so that folds and sagging are more evident, a process that is further amplified by photodamage in sun-exposed skin. Topical treatments are an important first line of therapy. TransFORM Body Treatment (TFB) with TriHex Technology (Alastin Skincare, Inc., Carlsbad, CA) has been developed to be used after procedures that breakdown subcutaneous fat, and additionally as a stand-alone treatment for skin crepiness and laxity.^1-4^ Clinical studies with TFB have shown histologic evidence of regenerated collagen and elastin.^2^ New collagen and elastin effectively reverse some of the aging processes and restore the youthful appearance of the skin. In addition, TFB is effective in “prejuvenation”—in other words, preventing signs of cutaneous aging as well as treating them after they have occurred. This study evaluated the topical use of TFB compared to a bland moisturizer, Cetaphil Lotion (Galderma, Fort Worth, TX), on both the extensor (sun-exposed) and volar (non–sun-exposed) sides of the forearms over a 3-month period evaluated by photographic changes, dermatopathology changes, cutaneous elasticity measurements using 2 separate methods, skin thickness, and patient-reported outcomes.

## METHODS

This prospective, randomized protocol was IRB approved by Veritas IRB Inc. (Montreal, Quebec, Canada). Eligible middle-aged male and female participants were without any skin disease, infection, or excessive skin laxity (Class 4 or 5 on the Rao-Goldman scale^5^). Participants with any history of abnormal skin reactivity to double-sided sticky tape or an allergy to the topical products were excluded. Participants who were pregnant, lactating, or planning on becoming pregnant during the study duration were also excluded. Eligible participants were consented and given a kit containing 2 identical looking bottles labeled right and left and instructed to apply to the designated forearm, twice daily. In each kit, TFB was randomly assigned to bottle right or left according to a random number table. After the morning application of the test and control articles, the provided SPF 30 sunscreen was applied to both arms. The study consisted of 4 visits: screening/baseline, and follow-up at weeks 4, 8, and 12. The study started in November 2019 and concluded in March 2020. At every visit the participants underwent the procedures detailed below.

### Photography

Photographs were taken at every visit with a LifeViz Micro 3D system (Quantificare Inc., Cumming, GA) and a Canon EOS1500D Rebel T7 camera (Canon USA Inc., Huntington, NY). A ruler was used at every visit to ensure the photograph was taken of the same location on the arm. Photographs were taken of the extensor and volar sides of both forearms.

### Elasticity Measurement Method 1

The first method used a Torsionometer (Carruthers Instruments, Vancouver, BC) which measures elasticity by twisting a section of skin and calculating the recoil velocity in degrees per second as the skin returns to its original position. The 25-mm diameter probe was attached to the skin with a double-sided adhesive, and then manually twisted 20° before being released. The recoil velocity was measured with an optical encoder and recorded onto a laptop which collected all the data. At every visit measurements were taken on clean, dry skin on the extensor and volar forearms. All measurements were performed in triplicate in the same location as indicated by a ruler and referencing the wrist crease as a datum.

### Elasticity Measurement Method 2

The second method used a DermaLab Combo (Cortex Technology, Hadsund, Denmark) to measure elasticity. The elasticity probe of this device is equipped with a suction chamber and double-sided adhesive tape is used to prevent folding of the skin under the edge surrounding the measurement chamber. The retraction time is the time it takes (in milliseconds) for the skin to retract to its original form from peak elevation. At every visit measurements were taken in triplicate on clean, dry skin on the extensor and volar sides of both forearms in the same location as indicated by a ruler and referencing the wrist crease as a datum.

### Ultrasound Measurements

An ultrasound probe DermaLab Combo (Cortex Technology, Hadsund, Denmark) was used to measure the skin thickness of the dermis in micrometers. The transducer was set to center frequency 20 MHz, bandwidth 5-35 MHz, focal distance 13 mm. A drop of water was applied to the skin prior to the measurement and the ultrasound measurement was taken in the same area of both the extensor and volar forearms at every visit.

### Biopsies

Two participants consented to having biopsies on volar and extensor surfaces of both arms prior to the use of the randomized topical products and post 12 weeks of application. The 4 biopsy sites were located in skin creases at the elbow in both extensor (sun-exposed) and volar (non–sun-exposed) skin of both arms. All biopsies were sent to an independent laboratory and evaluated by a blinded dermatopathologist. Several new histopathologic stains were used to detect changes in elastin and collagen content of the non–sun-exposed and sun-exposed skin.

### Patient-Reported Outcomes (PROs)

At each visit the participants were asked by the investigators to use the Rao-Goldman scale^5^ on each arm and complete a paper form (Appendix) to record their result (1, none; 2, shallow but visible; 3, moderately deep; 4, deep with well-defined edges; and 5, very deep with redundant folds).

## RESULTS

Nineteen participants enrolled in the study; however, only 13 (4 men, 9 women; mean age, 57 years; range, 38-74 years) completed the study in an average of 103 days (range, 97-126 days). All participants were indoor workers for their professional careers. Three participants had Asian type 3 skin, and 10 participants had Caucasian skin. Five participants (25%) were unable to complete their visits due to COVID-19 pandemic restrictions and 1 participant (5%) discontinued due to an erythematous scaly rash on the arm treated with TFB. There were no other adverse events reported.

### Photography

**Figure 1.**
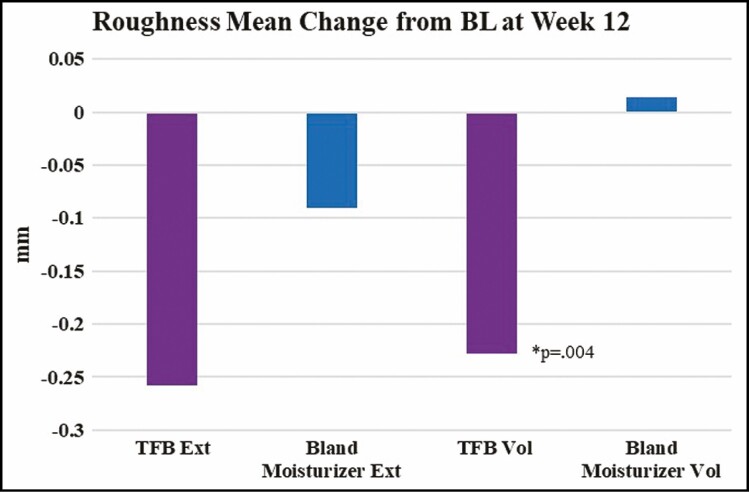
Photographic analysis of skin roughness quantifies the regularity on the skin surface. A decrease in roughness demonstrates less irregular, smoother skin.

**Figure 2.**
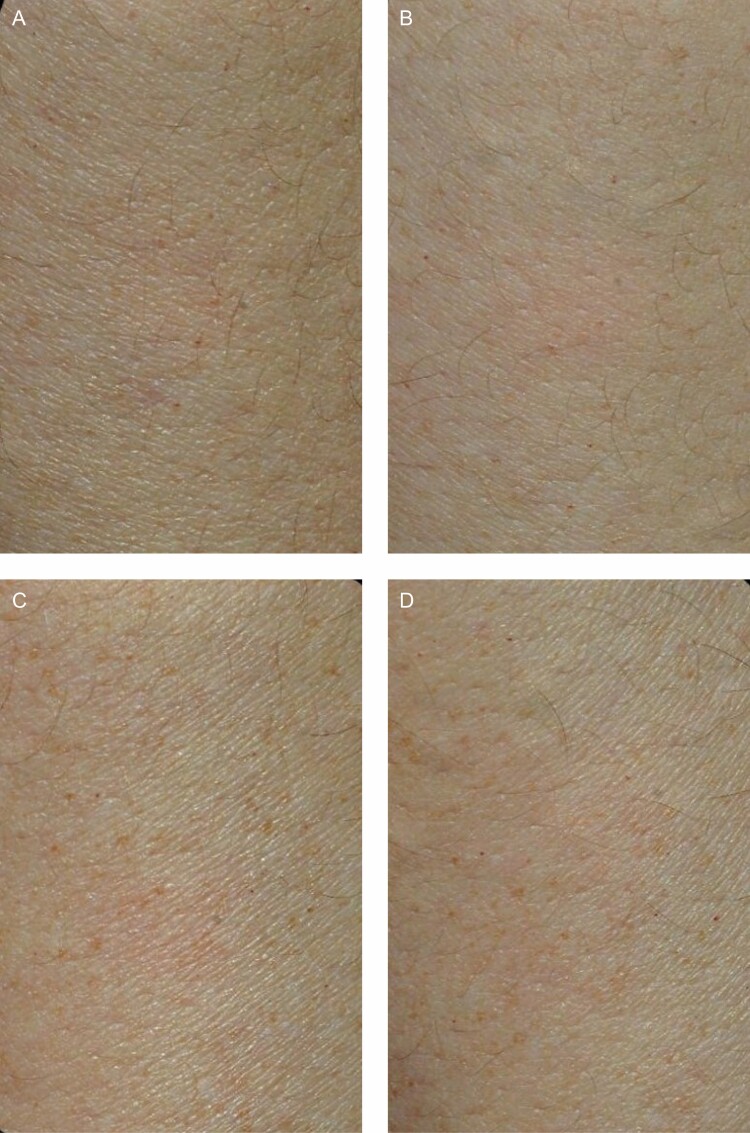
Quantificare microphotos for participant 2 (female, age 38 years): (A) TFB extensor forearm skin baseline; (B) TFB-treated extensor forearm skin at 3 months; (C) bland moisturizer extensor forearm skin baseline; (D) bland moisturizer–treated extensor forearm skin at 3 months. There is considerable visible smoothing of the skin surface and a marked diminution of the visible pores compared to the before-treatment photograph of the TFB side, more so than the bland moisturizer differences. This was confirmed by quantitative analysis performed with the Quantificare software. TFB, TransFORM Body Treatment.

A roughness analysis was performed on the 3-dimensional (3D) photographs, comparing week 12 to baseline. Roughness is a measurement of texture or regularity on the skin surface expressed in millimeters; a decrease in roughness is indicative of a reduction in variation which translates into smoother skin texture. The mean change in roughness from baseline to 3 months was compared between TFB and bland moisturizer arms. On the extensor side the change in roughness was –0.26 mm for TFB and –0.09 mm for bland moisturizer (*P* = 0.174). On the volar side the change in roughness was –0.23 mm for TFB and 0.01 mm for bland moisturizer (*P* = 0.004). Negative numbers indicate a change towards smoother skin at the end of the study (Figures 1 and 2).

### Elasticity Measurement Method 1

Measurements with the Torsionometer demonstrated a trend toward slower recoil velocity over the 12-week study for all measurement sites. The mean change in Torsionometer recoil velocity for the volar side was –56°/sec for bland moisturizer and –24°/sec for TFB (*P* = 0.61). The mean change in Torsionometer recoil velocity for the extensor side was –95°/sec for bland moisturizer and –63°/sec for TFB (*P* = 0.57). Negative numbers indicate a change to slower recoil velocities at the end of the study.

### Elasticity Measurement Method 2

**Figure 3.**
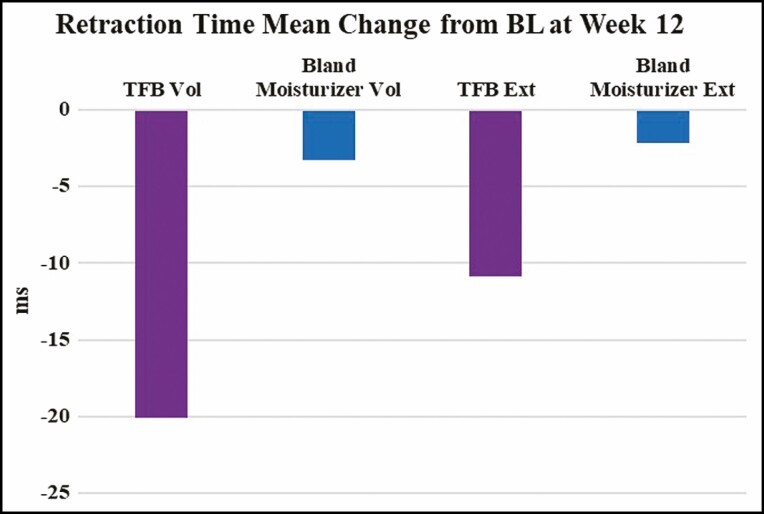
Change in retraction time compared with baseline (DermaLab Series, Cortex Technology), where a negative number indicates a faster retraction time.

The mean change from baseline was calculated for all Cortex measurements performed. The mean change in Cortex retraction time for the volar side was –3.25 ms for bland moisturizer and –20.08 ms for TFB (*P* = 0.33). The mean change in Cortex retraction time for the extensor side was –2.17 ms for bland moisturizer and –10.83 ms for TFB (*P* = 0.66). Negative numbers indicate a faster retraction time at the end of the study (Figure 3).

### Skin Thickness Measurements

**Figure 4.**
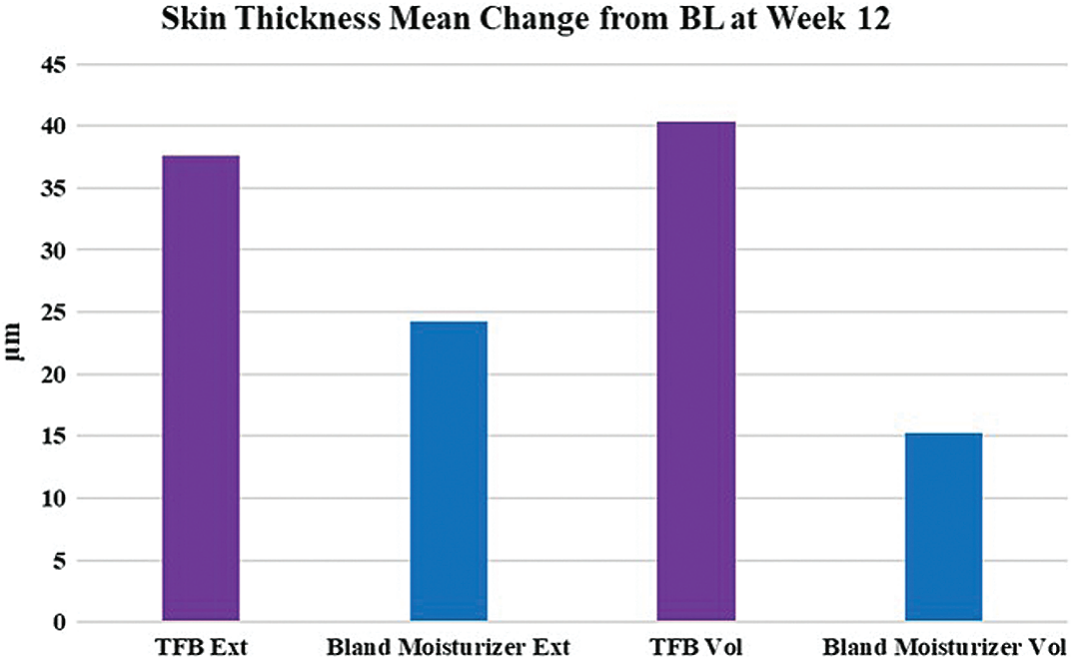
The mean change of skin thickness (in μm) from baseline as measured by ultrasound.

The mean change in skin thickness on the volar side was 15.3 μm for bland moisturizer and 40.4 μm for TFB (*P* = 0.44). The mean change in skin thickness on the extensor side was 24.2 μm for bland moisturizer and 37.6 μm for TFB (*P* = 0.74) (Figure 4).

#### Biopsies

**Figure 5.**
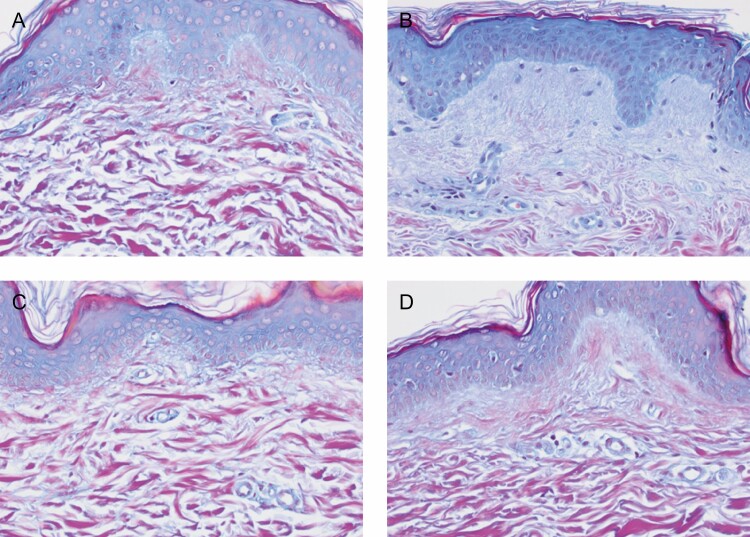
Volar side of the arm for participant 4 (female, age 50 years). The Herovici stain differentiates between young and mature collagen. Young collagen is stained blue whereas mature collagen is magenta. (A) Baseline TFB; (B) 3 months TFB; (C) baseline bland moisturizer; (D) 3 months bland moisturizer. At the end of the study there was more new, young collagen in the TFB side (B) than in the bland moisturizer side (D). TFB, TransFORM Body Treatment.

**Figure 6.**
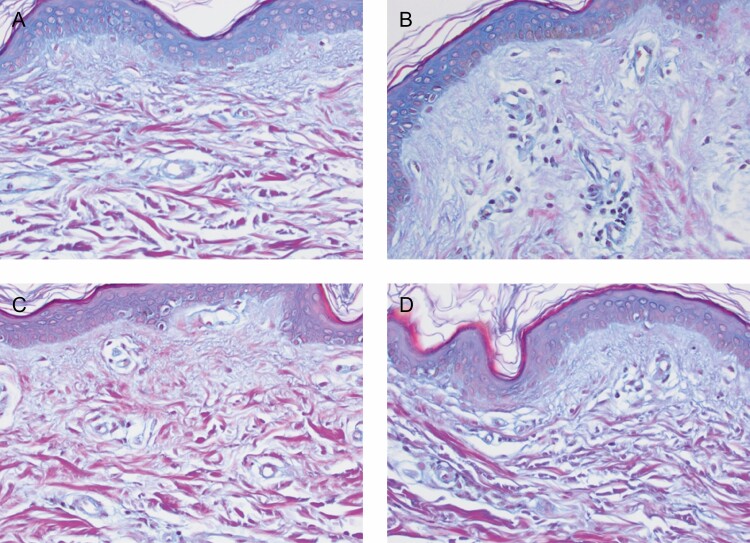
Volar side of the arm for participant 5 (female, age 52 years). (A) Baseline TFB; (B) 3 months TFB; (C) baseline bland moisturizer; (D) 3 months bland moisturizer. At the end of the study there was more new, young collagen in the TFB side (B) than in the bland moisturizer side (D). TFB, TransFORM Body Treatment.

**Figure 7.**
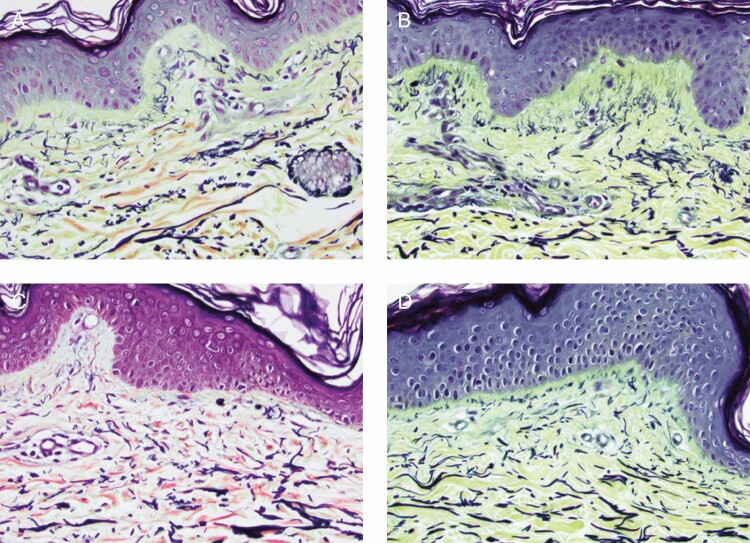
Volar side of the arm for participant 4 (female, age 50 years). The Movat stain is a pentachrome stain in which elastin stains black and collagen stains yellow. (A) Baseline TFB; (B) 3 months TFB; (C) baseline bland moisturizer; (D) 3 months bland moisturizer. There is more new elastin seen, particularly in the papillary dermis, of the volar skin of the TFB side (B) than the volar skin of the bland moisturizer side (D). Studying the Grenz zone between epidermis and dermis shows the vertically arranged elastic fibers extending from papillary dermis to epidermis. There is much more new elastin seen in the skin of the TFB side (red oval) than in the bland moisturizer side (yellow oval). TFB, TransFORM Body Treatment.

**Figure 8.**
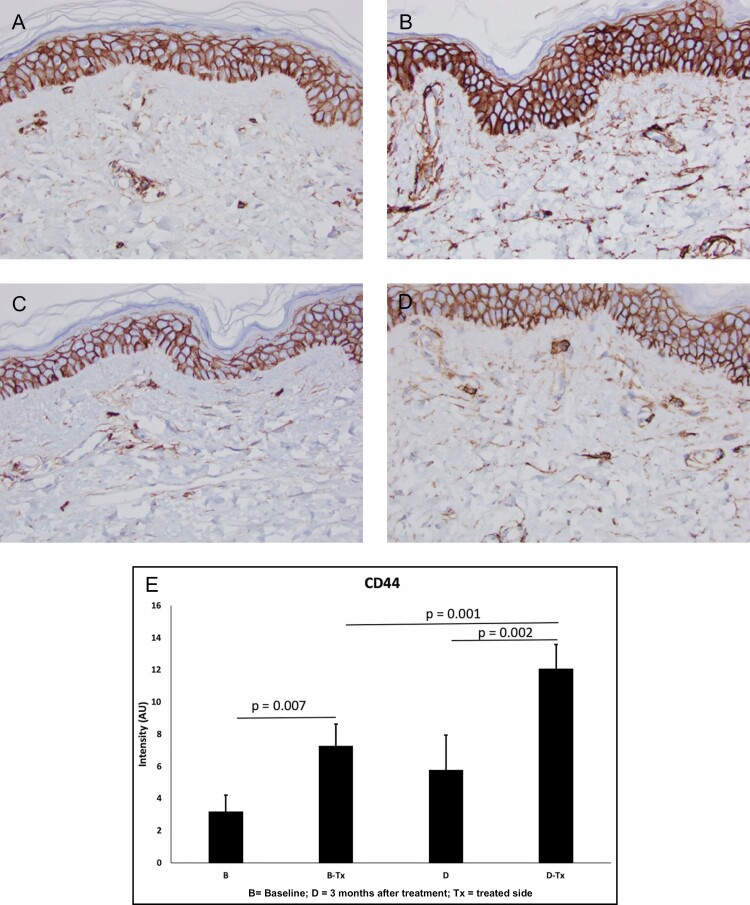
CD44 stain for participant 4 (female, age 50 years). (A) Baseline TFB; (B) 3 months TFB; (C) Baseline bland moisturizer; (D) 3 months bland moisturizer. There is more hyaluronan seen in the TFB arm (B) than in the bland moisturizer arm (D). (E) The images obtained after the CD44 immunohistochemical analysis were analyzed with ImageJ software. Five fields of view were assessed for each condition (at 10×). The mean intensity (in arbitrary units, AU) and standard deviation were determined for each condition. The values were compared with a Student’s *t* test; *P* < 0.05 was considered significant. CD44 expression significantly increased after both the bland moisturizer and TFB treatments. However, the intensity of CD44 after the TFB treatment was significantly greater than after the bland moisturizer treatment (168%; *P* < 0.01). These data indicate that the TFB treatment stimulates protein expression, potentially leading to hyaluronic acid signaling to a significantly greater degree compared with bland moisturizer. TFB, TransFORM Body Treatment.

Biopsies from the TFB-treated arm showed an increase in the mucopolysaccharide content, demonstrating new collagen formation and an increase in elastin fibers. Herovici stain demonstrated the increased mucopolysaccharides which represented new collagen formation primarily in the papillary dermis. This was evident in both participants’ biopsies with marked increases evident on the TFB-treated side in comparison with the bland moisturizer. The most notable changes occurred in the non–sun-exposed volar sides of the arm on the TFB sides in both subjects. Similarly, changes on the TFB volar side for elastin regeneration and hyaluronic acid receptor CD44 stain in the epidermis were evident in both subjects. The stains used were Movat stain to demonstrate very early elastin fiber formation on the papillary dermis, and CD44 stains which identify hyaluronic acid receptors (CD44) on the surface of epidermal cells in particular and to a lesser extent dermal extracellular matrix deposition (Figures 5-8).

Biopsies were all sent to a specialized histopathology laboratory; all samples were processed at the same time. Although quantification of the observations could have been made with techniques previously described in the literature,^6^ the differences were deemed to be obvious to the naked eye, rendering quantification unnecessary.

1. Herovici stain detects an increase in mucopolysaccharides which represents new collagen formation primarily in the papillary dermis (Figures 5 and 6). New collagen formation is clearly visible in the papillary dermis of the TFB group, to a much larger extent than in the bland moisturizer group.
2. Movat stain detects new elastin, also particularly seen in the papillary dermis as fine new threads extending perpendicular to the linear flow of the collagen fibers and extending to the dermoepidermal junction (Figure 7). Movat stains demonstrated new fine elastin fibers forming in the papillary dermis of the TFB group. Minimal neoelastogenesis was observed in the bland moisturizer group.
3. CD44 stain detects hyaluronic acid receptors which are associated with the formation of new hyaluronic acid (Figure 8). The images obtained after CD44 immunohistochemical analysis were analyzed with ImageJ software^7^ to assess the differences in stain intensity (Figure 8E). The data indicate that the TFB treatment stimulates protein expression, potentially leading to hyaluronic acid signaling to a significantly greater degree compared with bland moisturizer.

### PROs

The mean change in Rao-Goldman score from baseline to 3 months was calculated for both the volar and extensor sides, and results were compared between TFB and bland moisturizer. The mean change in Rao-Goldman score for the volar side was –0.17 for bland moisturizer and –0.33 for TFB (*P* = 0.25). The mean change in Rao-Goldman score for the extensor side was –0.08 for bland moisturizer and –0.17 for TFB (*P* = 0.36). In both cases there was smoothing perceived, but more so on the TFB side.

## DISCUSSION

Ninety percent of skin aging is due to sun damage.^8^ We thus wanted to compare the effects of a benign moisturizer and TFB on sun-damaged (extensor) arm skin and volar (non–sun-damaged skin). The arm seemed to be an excellent model, particularly as subjects could easily separate the treated areas and were not unhappy to have biopsies taken in the cosmetically hidden skin of their elbow creases. TFB has been shown through clinical studies and histology to increase collagen and elastin. Most recently, TFB has shown extracellular remodeling through gene expression studies.^9^ Therefore, TFB was used in this study to further characterize cutaneous elasticity,^10^ thickness, and histologic changes within the dermis in comparison to widely available bland moisturizer.

3D photography was used for a qualitative and semiquantitative evaluation of the skin changes over time. Visual evaluation of the photographs indicated that skin did appear smoother by the end of the study, particularly on the TFB side. A roughness measurement of the 3D photographs was performed with Quantificare analysis software (L Buchner, personal communication, August 18, 2020). On the extensor side of the arm both TFB-treated and bland moisturizer–treated skin became smoother over the course of the study, but the change on the TFB side was as much as 3 times that of the bland moisturizer side. On the volar side the difference between treatments was even more dramatic: although the TFB side became smoother, the bland moisturizer side essentially did not change (a change of –0.23 mm vs 0.01 mm, respectively; *P* = 0.004). The fact that the bland moisturizer had no impact on the volar side and some impact on the extensor side may be an indication that the hydrating properties of the moisturizer impacted the sun-damaged side of the arm more than the less-damaged volar arm.

**Figure 9.**
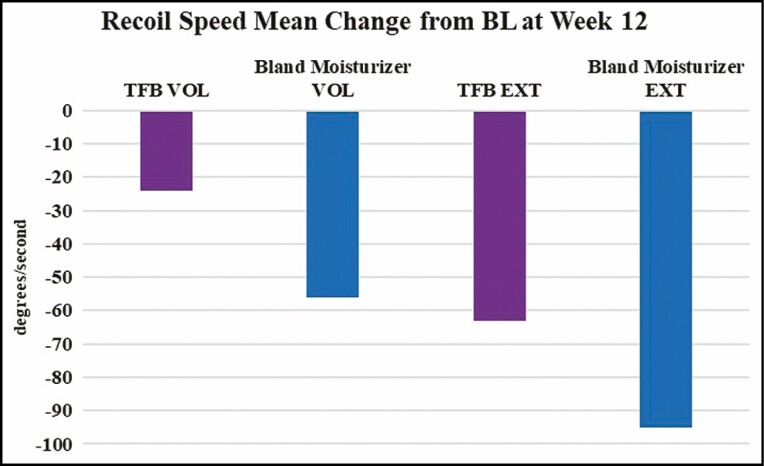
A negative change in recoil speed indicates that the skin was slower to return to its original shape at the end of the study. The bland moisturizer arm showed more slowing than the TransFORM Body Treatment arm.

The 2 devices measured a proxy for skin elasticity by different methods, namely retraction speed (Cortex Technology) and recoil velocity (Torsionometer). Although trends were seen, neither method was able to make a statistically significant differentiation between the TFB and bland moisturizer sides. Interestingly, the Cortex instrument showed a general trend of a shorter retraction time on both the TFB and bland moisturizer arms, whereas the Torsionometer showed a general trend of a slower recoil velocity on the TFB and bland moisturizer arms. In other words, on average, the Cortex recorded an 8-mm circle of deformed skin snapping back to its original shape faster at 12 weeks compared with baseline (volar: –3.25 ms for bland moisturizer and –20.08 ms for TFB; extensor: –2.17 ms for bland moisturizer and –10.83 ms for TFB), whereas on average, the Torsionometer recorded a larger 25-mm circle of skin twisting back to its original shape at a slower rate at the end of the study than at baseline (volar: –56°/sec for bland moisturizer and –24°/sec for TFB; extensor: –95°/sec for bland moisturizer and –63°/sec for TFB) (Figure 9).

**Figure 10.**
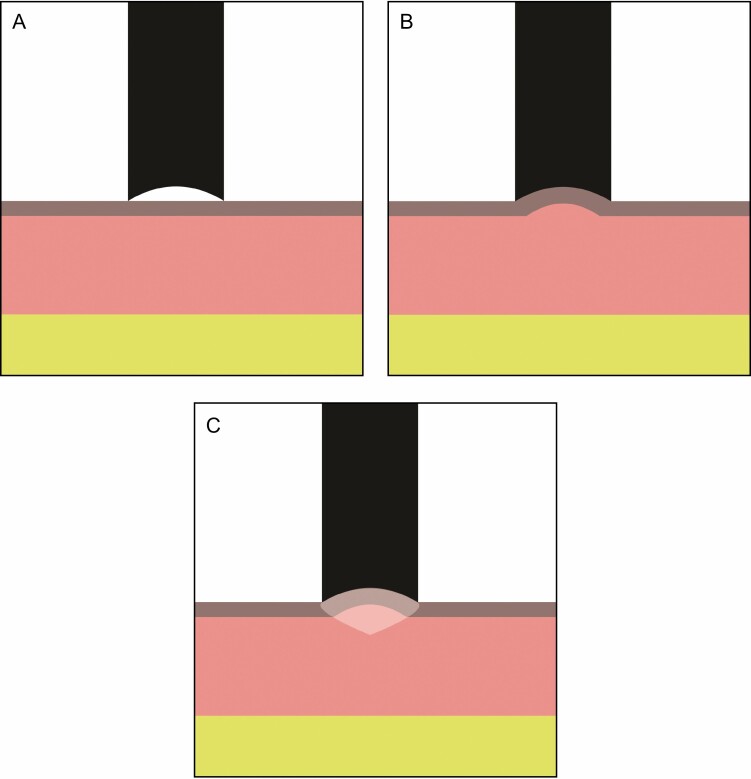
The Cortex device has an 8-mm diameter probe (A). During the measurement it sucks an 8-mm diameter portion of skin into the dome (B), then measures the time the skin takes to retract to its original position (ms). The depth and volume of impacted tissue is minimal, as shown by the shaded area (C).

**Figure 11.**
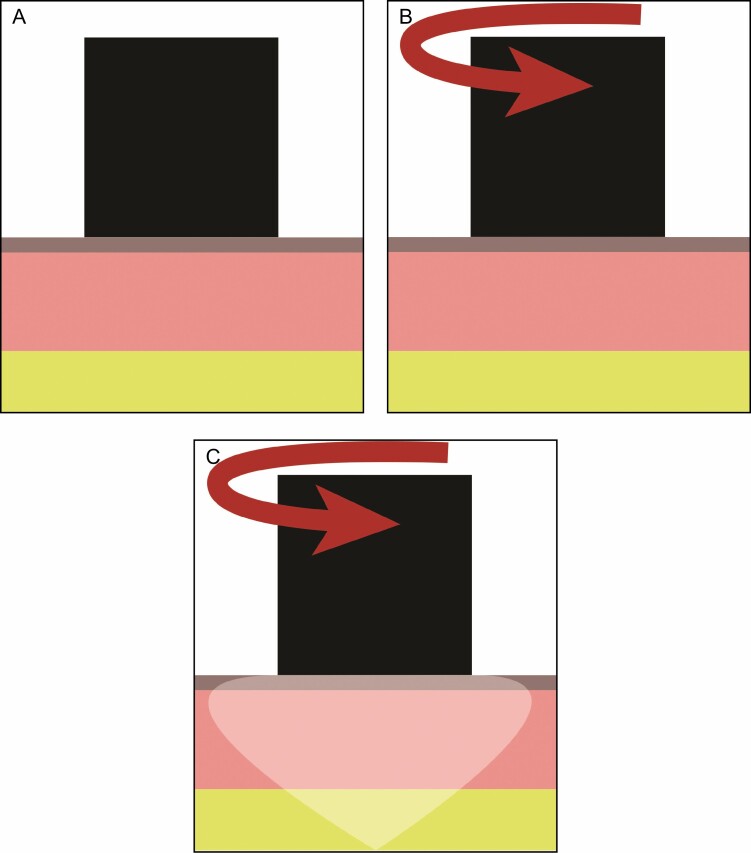
The Torsionometer has a 25-mm probe. (A) The device twists a 25-mm diameter portion of skin (B), then measures the rate at which the skin recoils to its original position (degrees/sec). The area, volume, and depth of tissue impacted is greater than with the Cortex device, as shown by the shaded area (C).

The trends shown by the Cortex instrument, although not statistically significant, are in accord with the quantitative observations of the histology and photography. However, the Torsionometer results seem to indicate the opposite trend: the skin recoiled slower instead of faster at the end of the study. This apparent contradiction could be explained by the manner in which the skin is distorted by the 2 measurement techniques, as illustrated in Figures 10 and 11. As can be seen, the Torsionometer measurement involves a greater area, depth, and volume of tissue than the Cortex, and therefore results from the 2 techniques cannot be directly compared.

We propose the concept of “cutaneous resistance” (CR) which is the reluctance of the skin to return to its original shape after deformation. In the context of the Torsionometer, skin elasticity (SE) is the force that returns the probe to its original position, whereas CR can be viewed as a damping force that slows the movement of the probe. Therefore, the recoil speed (RS) is proportional to CR + SE. We hypothesize that whereas the Cortex measures the SE of a shallow portion of the skin, the Torsionometer measures the combined effect of SE and CR in a larger volume of the skin and subcutaneous fibroseptal network.

Skin is a viscoelastic material and we know that the aqueous component in both TFB and benign moisturizer treatments provided hydration, and therefore increased the weight and possibly also the viscosity of the skin. Increased weight and/or viscosity would presumably have a damping effect, effectively increasing CR. Figure 9 shows recoil speeds decreased for all arms—indicating increased CR—however, the magnitude of the change was smaller for the TFB arm. We reason that the increased collagen and elastin seen in the TFB-treated skin increased the SE, which partially counteracted the increased CR from the moisturizer effect. With this view, the Torsionometer results are consistent with the other observations in the study.

The trends shown in skin thickness were somewhat similar to those shown in the roughness measurements: all skin became thicker over the course of the study; however, there was a greater increase for the TFB-treated sides. Hydration of the skin may have had an impact on the thickness measurements.

Furthermore, histology of the 2 arms demonstrated improved collagenesis and elastogenesis on the TFB arm. In this case, the Herovici and Movat stains were used expressly for the purpose of identifying early new collagen (mucopolysaccharides) and new elastin which is very difficult to do with routine staining. Quantification could be arrived at by assessing the percentage magenta to blue conversion with Herovici stain and new vertical elastin fibers in papillary dermis with Movat stain. However, the changes were so obvious this was deemed unnecessary. A detailed description and clarification of the techniques has recently been published.^6^ ImageJ analysis completed for the CD44 stains demonstrated a statistically significant increase in intensity on the TFB arm. When considering all these results, it is interesting to note that although TFB resulted in markedly improved changes on extensor and volar sides compared with the bland moisturizer, the maximum differences between the groups was evident on the non–sun-exposed areas. We see the desired improvements in sun-exposed areas, but one could speculate that the nondamaged regenerative reserve (more efficient cellular signaling) that is present in non–sun-exposed areas allows dramatic changes to take place when challenged with the correct active ingredients. This was not so for the bland moisturizer and provides a possible validation of the “prejuvenation” concept of stimulating regenerative changes before excess damage has occurred. This is also particularly apt for a body product which is often used in these non–sun-exposed areas.

### Prejuvenation

The concept of prejuvenation, which is “treatment to prevent the appearance of aging,” has gained traction in recent years. First discussed by Arndt in 2013, the concept recognizes skin aging not only as a treatable condition after the fact, but indeed as a preventable condition with appropriate preventative treatments before the aging changes are permitted to occur.

Dermatologists and plastic surgeons have for years taught our communities about prevention of sun exposure, daily year-round topical sunscreen, and sun-protective clothing. They have also strongly suggested this behavior begin in childhood and continue through life on the basis that “the earlier these treatments start, the more effective these treatments will be.” ^11^

Our small, prospective, randomized study has shown that treatment of the non–sun-exposed inner arm with TFB is much more effective in producing more new collagen and elastin with improved visual texture and calculated reduction in roughness than is seen on the equally treated sun-exposed extensor side and much greater than treatment on either side of the arm with a bland moisturizer.

This would suggest that early protective maintenance of skin collagen and elastin with topical stimulatory products such as TFB could be an important addition to cornerstone treatment of already photodamaged skin, thus maintaining healthy and youthful skin throughout life.

Our study showed that regenerative changes occur to a much greater extent in non–sun-exposed skin than in sun-exposed skin. This information supports the concept of “prejuvenation” ^11,12^ where regenerative changes are stimulated before excess damage has occurred.

The changes in PROs were a limitation to the study as they were not statistically significant, which suggested that patients did not perceive the structural changes that the other outcomes detected. However, the reported outcomes did show trends that were consistent with the other outcomes. Patients reported a slight reduction in wrinkling on the arm treated with bland moisturizer, and a more noticeable reduction in wrinkling in the TFB-treated arm. It is possible that statistical significance was not achieved due to the small study population or because the 5-point Rao-Goldman scale was not sensitive/accurate enough for patients to differentiate the changes from the start to the end of the study.

Bland moisturizer was selected as the control with the understanding that it would likely not cause any structural changes to the skin. However, it appeared to have some effect in the increased moisture content of the skin, and therefore impacted outcomes. It seems from roughness, skin retraction time, skin recoil velocity, skin thickness, and PROs that there was an observable impact from bland moisturizer. We would expect this impact to be transient, whereas the impact of the TFB was shown by 3D photography and confirmatory histology to be structural and therefore more enduring.

Although the quantitative data showed trends that agreed with the qualitative observations, statistical significance was not reached for most observations. The small size of the study, which was further exacerbated by a premature end to the study due to COVID restrictions, was a limitation that may have impacted our conclusions.

## CONCLUSIONS

As judged by photography, histology, and PROs, TFB has been shown to be more effective than bland moisturizer in preventing as well as treating aging/sun-damaged skin and has a pronounced effect on the production of new collagen and elastin. Efforts to quantify the changes based on measurements of skin thickness, skin retraction speed, and skin recoil velocity showed trends that agree with the visual data. TFB has a more a pronounced effect on the production of new collagen and elastin in non–sun-exposed skin vs sun-exposed skin, as seen with 3D photography and histopathology. This supports the recent concept of “prejuvenation”—that the photoprotective signaling mechanism in non–sun-exposed skin is still intact, which allows it to thicken from within by producing more collagen, elastin, and hyaluronic acid when stimulated with the correct topical formulation.

## Supplementary Material

sjab161_suppl_Supplementary_Appendix
