# Supplementary figures and images for: Prospective, Randomized, Comparative Study of the Cutaneous Effects of a Topical Body Treatment Compared to a Bland Moisturizer
Source: Aesthet Surg J. 2021 Apr 2;41(9):NP1188–98. doi: 10.1093/asj/sjab161 (PMC8361352; doi:10.1093/asj/sjab161)

Patient-Reported Outcome Forms


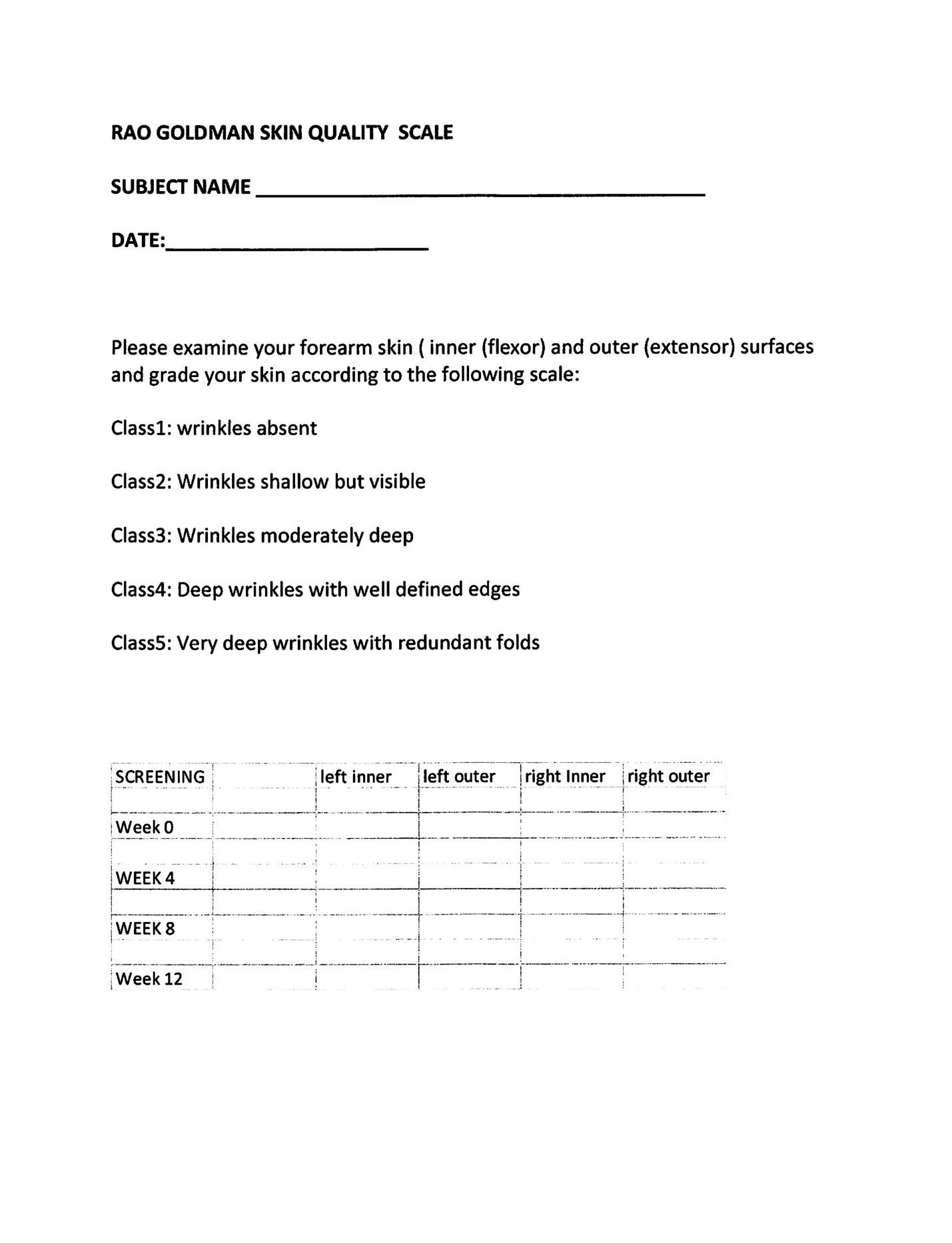


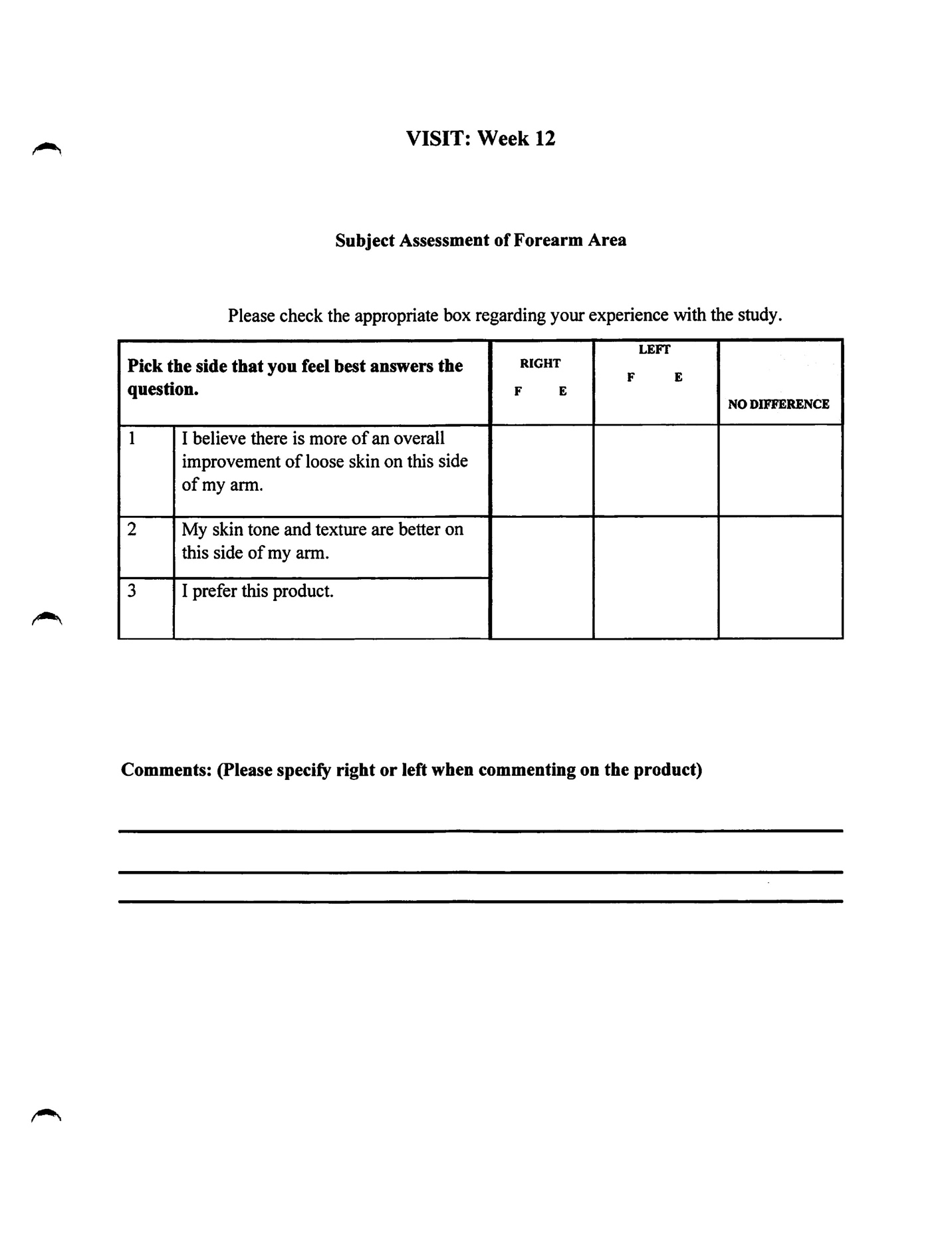

Supplement: sjab161_suppl_Supplementary_Appendix [file sjab161_suppl_supplementary_appendix.docx]
